# Fully hydrogenated canola oil extends lifespan in stroke-prone spontaneously hypertensive rats

**DOI:** 10.1186/s12944-021-01540-7

**Published:** 2021-09-12

**Authors:** Kenjiro Tatematsu, Daisuke Miyazawa, Yoshiaki Saito, Harumi Okuyama, Naoki Ohara

**Affiliations:** 1 Department of Radiochemistry, Gifu Pharmaceutical University, 5-6-1 Mitahora-higashi, Gifu, Gifu-ken, Japan; 2 Open Research Center for Lipid Nutrition, Kinjo Gakuin University College of Pharmacy, 2-1723 Omori, Moriyama-ku, 463-8521 Nagoya, Aichi Japan; 3 Department of Pathology, Food and Drug Safety Center, Hatano Research Institute, 729-5 Ochiai, 257-8523 Hadano, Kanagawa Japan

**Keywords:** Fully hydrogenated canola oil, SHRSP rats, Survival, Blood pressure, Renal dysfunction

## Abstract

**Background:**

Canola oil (Can) and several vegetable oils shorten the lifespan of stroke-prone spontaneously hypertensive rats (SHRSP). Although similar lifespan shortening has been reported for partially hydrogenated Can, the efficacy of fully hydrogenated oils on the lifespan remains unknown. The present study aimed to investigate the lifespan of SHRSP fed diets containing 10 % (w/w) of fully hydrogenated Can (FHCO) or other oils.

**Methods:**

Survival test: Upon weaning, male SHRSP were fed a basal diet for rodents mixed with one of the test oils —i.e., FHCO, Can, lard (Lrd), and palm oil (Plm) throughout the experiment. The animals could freely access the diet and drinking water (water containing 1 % NaCl), and their body weight, food intake, and lifespan were recorded.

Biochemical analysis test: Male SHRSP were fed a test diet with either FHCO, Can, or soybean oil (Soy) under the same condition, except to emphasize effects of fat, that no NaCl loading was applied. Soy was used as a fat source in the basal diet and was set the control group. Blood pressures was checked every 2 weeks, and serum fat levels and histological analyses of the brain and kidney were examined after 7 or 12 weeks of feeding.

**Results:**

During the survival study period, the food consumption of FHCO-fed rats significantly increased (15–20 % w/w) compared with that of rats fed any other oil. However, the body weight gain in the FHCO group was significantly less (10–12 %) than that in the control group at 9–11 weeks old. The FHCO (> 180 days) intervention had the greatest effect on lifespan, followed by the Lrd (115 ± 6 days), Plm (101 ± 2 days), and Can (94 ± 3 days) diets. FHCO remarkably decreased the serum cholesterol level compared with Can and the systolic blood pressure from 12 to 16 weeks of age. In addition, while some rats in the Can group exhibited brain hemorrhaging and renal dysfunction at 16 weeks old, no symptoms were observed in the FHCO group.

**Conclusion:**

This current study suggests that complete hydrogenation decreases the toxicity of Can and even prolongs the lifespan in SHRSP.

**Supplementary Information:**

The online version contains supplementary material available at 10.1186/s12944-021-01540-7.

## Background

Dietary fat intake can promote or prevent chronic disease [1, 2]. It was reported that the addition of canola oil (Can) to standard rat chow at 10 % (w/w) shortened the lifespan of stroke-prone spontaneously hypertensive rats (SHRSP) compared with those fed soybean oil (Soy) [3–6]. Shortened lifespan were also observed with several edible oils [4, 5]. Phytosterols were inferred to be causative for this lifespan-shortening since they are readily accumulated in bodies of SHRSP, probably due to a mutation in the ATP-binding cassette transporter G5/8 (*abcg5/8*) [7–10]. However, several oils with low levels of phytosterols also shorten the lifespan, and supplementation of Can with phytosterols does not impact the lifespan [10, 11]. Hence, the determinative substance for lifespan shortening remains unknown. In addition to the lifespan shortening, Can in SHRSP causes renal dysfunction [12], decreases blood cell membrane deformability [7], increases blood pressure, inhibits the Na^+^/K^+^–ATPase function [13], suppresses the reproductive activity [14], perturbs steroidal hormone levels [15], and increases oxidative stress [16–18]. Furthermore, these effects of Can have been reported in rats of other strains and in other animal species [19–23]. SHRSP are an animal model comparable to hypertension with cerebral stroke in humans [24]. These rats have a higher risk of lethal cerebral stroke under sodium chloride (NaCl) loading from drinking water, possibly because of the abnormally elevated blood pressure (220–240 mmHg) induced by renal dysfunction. However, whether Can and other edible oils affect the blood pressure in SHRSP remains unclear [12]. Because SHRSP exhibit symptoms of metabolic syndrome [25, 26], variations in dietary fat could alter their lipid metabolism [12]. Thus, it is possible that the lipid level affects the progression of genetic disease in SHRSP.

Partially hydrogenated vegetable oils with higher melting points are used in margarine preparation or shortening. However, the hydrogenation process may generate toxic trans-fatty acids (FAs) from the hydrogenation of unsaturated FAs and may be associated with cardiovascular disease [27] and inflammatory and oxidative stress [28]. This problem could be circumvented by complete hydrogenation converting all unsaturated FAs, including *trans* FA, to saturated FA (SFA). For instance, inter-esterification of oils with fully hydrogenated FA produces margarine without *trans* fat [29]. The consumption of fully hydrogenated edible oils is anticipated to increase as consumers become more conscious of the health risks associated with consuming *trans* FA. Reportedly, partially hydrogenated Soy shortens the lifespan in SHRSP, although original Soy does not [30]; this finding implies the production of substances toxic to SHRSP in the hydrogenation process. Although the safety of fully hydrogenated edible oil has been previously investigated [31], the efficacy of fully hydrogenated Can (FHCO) in SHRSP survival remains unclear. Hence, for the first time, the current study aimed to investigate the effects of fully hydrogenated oil on the lifespan and biological parameters of SHRSP to establish the safety of edible oils with high-melting-point.

## Methods

### Diet and animals

In this study, all animal experiments were performed in accordance with the National Institutes of Health guidelines on animal care [32]. The study protocol was approved by the Institutional Animal Care and Use Committee of Gifu Pharmaceutical University (Gifu, Japan; Permit nos. 2011 − 382 and 2012-099) and the Committee for Animal Research and Welfare of Gifu University (Permit nos. 23–55 and 24–77). All rats were maintained under specific pathogen-free conditions at a temperature of 23 °C ± 3 °C and humidity of 55 % ± 15 % in a 12:12-h light/dark cycle.

The basal conventional diet (CE-2; Clea Japan, Tokyo, Japan) contained soybean meal, fish meal, skimmed milk, Soy, corn, wheat, wheat bran, alfalfa meal, vitamins, and minerals; the proportion of endogenous FA was 2.7 %. The experimental diets were prepared by mixing CE-2 with each oil at a ratio of 9:1 in a weight base. The diet foods were stored at − 20 °C for < 3 months before serving. Of note, all vegetable oils were commercially available for human consumption and were purchased from local markets. In addition, FHCO (melting point, 92 °C) is a product for human consumption. The test diet was replaced every 2 days.

SHRSP were obtained from the Disease Model Cooperative Research Association (Kyoto, Japan).

### Survival test

In the survival experiment, 80 male SHRSP without any difference in the body weight were assigned randomly to 4 different dietary groups, and animals in each group were fed 1 of the test diets beginning at the age of 4 weeks. In addition, rats were provided with NaCl (drinking water containing 1 % NaCl) and fed a diet containing 10 % w/w Can, lard (Lrd), palm oil (Plm), or FHCO. Tables 1 and 2 present the FA compositions and sterol contents of the diets.

**Table 1:** The FA composition of test diets (%)

FA	FHCO	Lrd	Plm	Can	Soy	CE-2
14:0	0.3	1.4	1.0	0.3	0.2	0.2 ± 0.1
16:0	9.4	25.4	28.8	8.3	14.4	14.3 ± 0.1
16:1	0.3	1.8	0.5	0.5	0.4	0.8 ± 0.0
18:0	66.1	12.7	2.9	2.0	5.1	13.9 ± 0.2
18:1	7.6	36.8	42.4	52.9	22.9	19.9 ± 0.1
18:2 n-6	12.8	18.8	21.9	26.1	50.0	40.5 ± 0.1
18:3 n-3	0.8	1.2	1.0	7.2	5.3	3.1 ± 0.0
20:0	1.4	0.2	0.3	0.5	0.3	0.1 ± 0.1
20:1	0.2	0.7	0.4	1.1	0.4	0.8 ± 0.1
20:5 n-3	0.4	0.5	0.5	0.5	0.5	1.3 ± 0.0
22:0	0.3	n.d.	0.1	0.3	0.2	0.2 ± 0.0
22:6 n-3	0.3	0.4	0.4	0.4	0.4	0.9 ± 0.0
n-6/n-3	8.2	9.1	11.5	3.2	8.1	7.6
Total FA (mg/100 mg diet)	13.8	13.1	14.7	14.0	14.1	5.3 ± 0.3

Test diets were prepared by mixing a conventional diet and edible oils at a 9:1 ratio. CE-2 data are represented as the mean ± SD (*n* = 3)

Elaidic acid (*trans* 18:1) was not detected in all diets

Abbreviations: *Can* canola oil, *CE-2* basal conventional diet, *FA* fatty acid, *FHCO* fully hydrogenated canola oil, *Lrd* lard, *n.d.* not detected, *Plm* palm oil, *SD* standard deviation, *Soy* soybean oil

**Table 2:** Sterol levels of test diets (mg/g diet)

Sterol	FHCO	Lrd	Plm	Can	Soy	CE-2
Cholesterol	75.5	79.1	70.2	76.6	78.7	81.6 ± 1.7
Brassicasterol	5.8	n.d.	n.d.	6.9	n.d.	n.d.
Campesterol	47.3	14.6	11.9	38.9	21.8	14.6 ± 1.2
Stigmasterol	8.8	5.7	4.0	7.8	13.6	5.4 ± 0.1
β-Sitosterol	94.4	44.8	32.3	91.2	71.5	33.9 ± 2.0
Total phytosterol	156.3	65.1	48.2	144.8	106.9	53.8 ± 3.0
Total sterol	231.8	144.2	118.4	221.4	185.6	135.5 ± 4.7

Data of CE-2 are represented as the mean ± SD (*n* = 3)

Abbreviations: *Can* canola oil, *CE-2* basal conventional diet, *FHCO* fully hydrogenated canola oil, *Lrd* lard, *n.d. *not detected, *Plm* palm oil, *SD* standard deviation, *Soy* soybean oil

All animals were allowed free access to food and drinking water. Animals’ body weight and food intake were monitored weekly until the age of 11 weeks. Although the survival time was determined under the specific pathogen-free conditions explained above, the mean survival time of a given dietary group varied markedly because of unknown factors. Thus, both the Can and Lrd groups for use in determining as controls and determined the lifespan shortening activity of the other groups. Animals with stroke-related symptoms and those who survived past the control lifespan were euthanized with an excess amount of pentobarbital (100–150 mg/kg, intraperitoneal). Of note, all animals were checked at least twice daily to determine the accurate survival time and were euthanatized as necessary.

### Biochemical analysis test

In addition to the survival test, another animal experiment was conducted to further investigate the effects of full hydrogenation on blood pressure, lipid levels in serum, and histological analyses. In this experiment, the Soy diet was used as a negative control, as in previous studies [14–18, 33]. Notably, Soy is the main fat source of CE-2, and no significant difference was found in survival times of SHRSP fed a 10 % Soy diet and original CE-2 (unpublished data). Salt loading through drinking water did not appear to impact the effect of any oil. For the biochemical analysis test, 36 male SHRSP were assigned and divided randomly into 3 groups. They were fed a diet containing either Can, Soy, or FHCO from 4 to 16 weeks old. This experiment aimed to compare the effects of oils on blood pressure, serum levels of cholesterol and triglyceride, and brain and kidney pathologies. Notably, NaCl loading was not performed to avoid the impact of the stroke-related condition, which might have interfered with the findings, and to precisely determine the differences in the efficacy of oils. In addition, body weight, food intake, and water intake were monitored weekly, and blood pressure was measured every 2 weeks with the tail-cuff method using the MK-2000ST blood pressure monitor (Muromachi Kikai Co., Tokyo, Japan). Then, half of the rats in each dietary group were sacrificed at 11 weeks old when they exhibited the pathology with NaCl loading, and the remaining rats were sacrificed at 16 weeks old under pentobarbital anesthesia (40 mg/kg, intraperitoneal). All serum samples were stored at − 80 °C until use; the brain and kidneys were removed and fixed in 10 % buffered formalin solution for histological examination.

### Lipid analysis

The FA composition and sterol content of test diets were determined as described previously, with some modifications [11]. Briefly, total lipids were extracted using the method proposed by Bligh and Dyer [34]. For FA analysis, the total lipid fraction was treated with 1.37 mol/L HCl in methanol (Tokyo Kasei, Tokyo, Japan) to convert FA to methyl esters. After extracting FA methyl esters with petroleum ether, FA levels were quantified by gas chromatography (GC) using a capillary column (DB-225; J&W Scientific, Folsom, CA) with heptadecanoic acid as an internal standard. For sterol analysis, total lipids were incubated with 10 % potassium hydroxide + ethanol solution at 100 °C for 2 h for saponification. Then, the sterol fraction was recovered with hexane and converted to trimethylsilyl derivative using TMS-HT® reagent (Tokyo Kasei). Next, sterol levels were estimated by GC (column, DB-1; J&W Scientific) using betulin as an internal standard. Furthermore, serum cholesterol and triacylglycerol (TG) levels were evaluated with the Cholesterol E-kit and Triacylglycerol E-kit, respectively (Wako Pure Chemical Industries, Osaka, Japan).

### Histological analysis

Brain and kidney tissue samples were fixed and embedded in paraffin, cut into 4-mm-thick coronal sections, and then stained with hematoxylin and eosin for histological examination. Pathologist compared each histological sample with normal tissues and determend a relative grade based on the 5-ranked score (–; No abnormal change, ±; very slight change, +; slight change, ⧺; moderate change, ⧻; marked change).

### Statistical analysis

Statistical analyses were performed using KyPlot v.2.0 software (Keyence, Osaka, Japan). Data are presented as mean ± standard error of the means (SEM; survival) or mean ± standard derivation (SD; other variables). Survival rates were analyzed with the log-rank test to assess differences in the late phases of survival curves and with the Wilcoxon signed-rank test (nonparametric) to assess differences in the early phase. In addition, a one-way analysis of variance (ANOVA) was performed followed by Tukey’s test, to compare multiple groups with respect to blood pressure (mmHg) and serum lipid levels (mg/mL). A two-way repeated measures ANOVA was performed, followed by Tukey’s test, to compare multiple groups with respect to age and either the body weight gain or food intake. If any interaction was observed, multiple comparisons were performed using Tukey’s test as a post hoc test for each week. and *P* value < 0.05 was considered statistically significant.

## Results

### Components of the test diets

Tables 1 and 2 present the FA compositions and sterol levels, respectively, of the test and conventional basal (CE-2) diets. The test diets were prepared from CE-2, and the required levels of linoleic acid were attained with 4.1 % crude oil in the basal diet. FHCO comprised three FAs: 5.3 % palmitic acid (16:0), 92.9 % stearic acid (18:0), and 1.8 % arachidic acid (20:0). FHCO was fully hydrogenated and contained no unsaturated FA, including *trans* FA. Thus, the FHCO diet contained > 75 % SFA and unsaturated FA derived from the basal diet. Similarly, the Lrd and Plm diets comprised similar FA compositions: 25.4–28.8 % 16:0, 36.8–42.4 % 18:1 [oleic acid], and 18.8–21.9 % 18:2n-6 [linoleic acid] except for 18:0, which was 12.7 % in the Lrd diet and 2.9 % in the Plm diet. The Can diet contained approximately 50 % 18:1, 25 % 18:2, and 7.2 % 18:3n-3 [α-linolenic acid], resulting in a low n-6/n-3 ratio.

Regarding sterol levels, the basal diet CE-2 contained adequate amounts of cholesterol (70–85 mg/g diet) and phytosterols (40–55 mg), which decreased the impact of differences between test oils. In addition, brassicasterol was detected in Can and FHCO but not in the other three oils. The total phytosterol levels were similar in Can and FHCO but slightly decreased in Soy. The phytosterol levels in the Lrd and Plm diets were negligible. Furthermore, the total sterol levels varied according to the phytosterol levels.

### Survival test

**Fig. 1:**
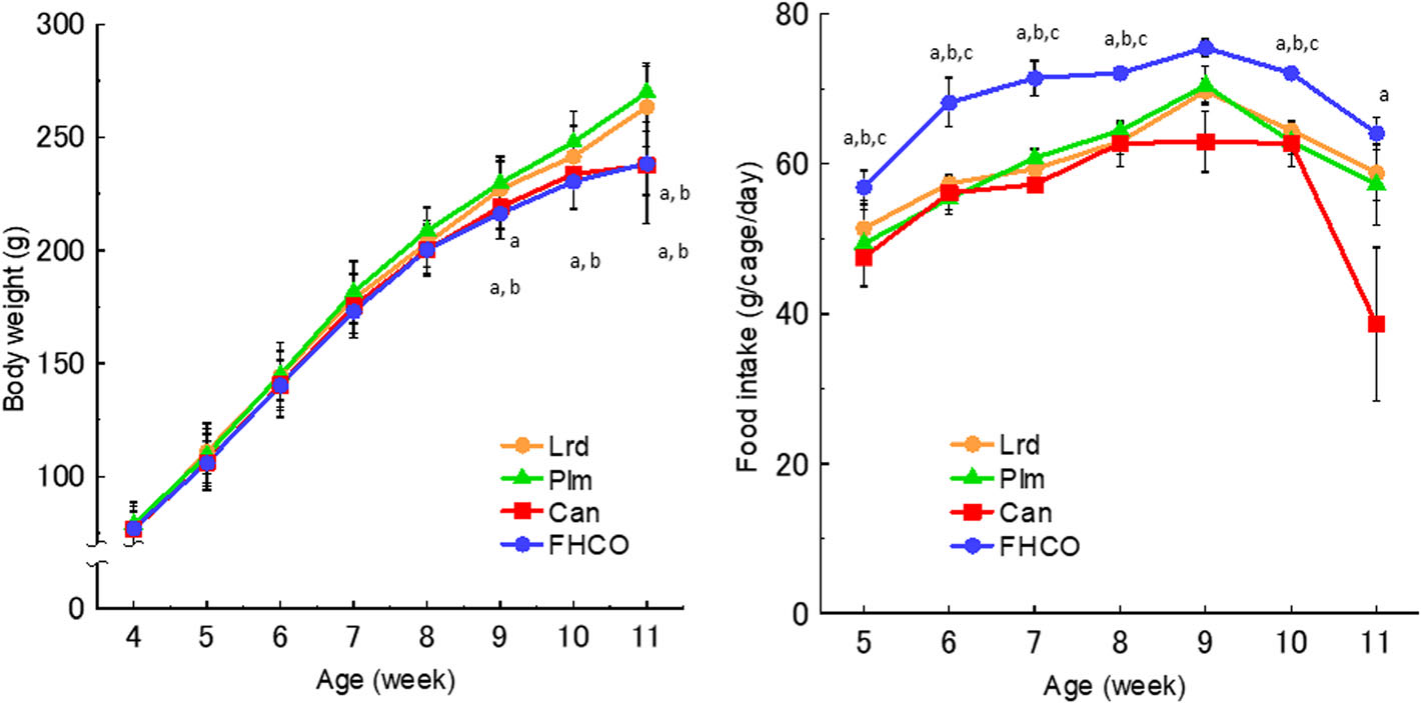
**a **Body weight and **b** food intake of SHRSP. Values represent mean ± SD (*n* = 20/group for body weight; *n* = 5 cages/group for food intake). ^a^*P* < 0.05 vs. Lrd group; ^b^*P* < 0.05 vs. Plm group; ^c^*P* < 0.05 vs. Can group. Abbreviations: Can, canola oil; FHCO, fully hydrogenated canola oil; Lrd, lard; Plm, palm oil; SHRSP, stroke-prone spontaneously hypertensive rat

To measure survival, SHRSP were fed the test diet for beginning at 4 weeks old (28 days). Changes in body weight and food intake were assessed in SHRSP fed a diet containing 10 % or either Can, Lrd, Plm, or FHCO from the age of 5 to 11 weeks (Fig. 1). The findings revealed that the interactions between the type of dietary oil and body weight gain and food intake were both significant. In the early period, the food intake was approximately 15–20 % higher in FHCO rats than in rats in the other three groups; this marginal increase persisted up to 10 weeks but disappeared at the age of 11 weeks. In addition, the body weight in the Can group was markedly decreased at the ages of 9 and 11 weeks, possibly reflecting a pathological state because of stroke. No significant differences in body weight were observed until the age of 8 weeks; however, the body weights of the FHCO and Can groups were decreased by approximately 10–15 % compared with the other two groups from the ages of 9 to 11 weeks. Starting at this time, severe symptoms of stroke, including visible bleeding, diarrhea, and paralysis, were observed in the Can group; these symptoms were later observed in the Lrd and Plm groups as well, but the FHCO group exhibited no signs of illness for the duration of the experimental period (Supplementary Table S1).

**Fig. 2:**
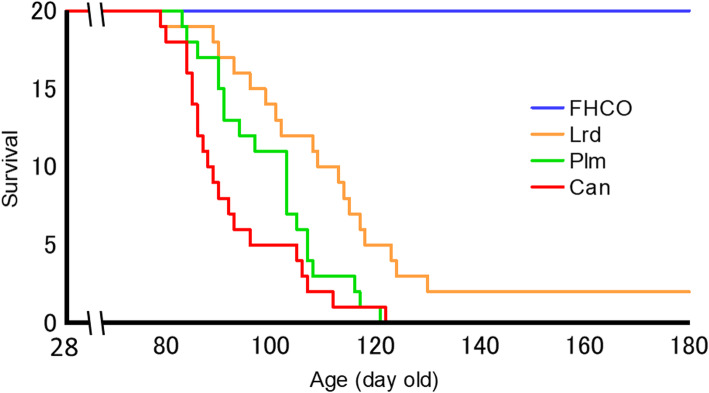
Survival curves of SHRSP fed test diets containing 10 % edible oils. Abbreviations: Can, canola oil; FHCO, fully hydrogenated canola oil; Lrd, lard; Plm, palm oil; SHRSP, stroke-prone spontaneously hypertensive rat

Lethal strokes occurred in rats starting at 81 days old, and more than half the rats died suddenly, especially in the early phase (Fig. 2; Table 3). The survival time (mean ± SEM) was shortest in the Can group (94 ± 3 days), followed by the Plm (101 ± 2 days) and then Lrd (115 ± 6 days) groups. The differences between the three groups were statistically significant (*P* < 0.05), except between the Lrd and Plm groups with the Wilcoxon signed-rank test and between the Can and Plm groups with the log-rank test (Table 3). None of the rats fed the FHCO diet died during the study period (> 180 days).

**Table 3:** Statistical analysis of the survival data

	Statistical test	FHCO	Lrd	Plm	Can
Survival (mean ± SEM)		> 180 ± 0	115 ± 6	101 ± 2	94 ± 3
vs. FHCO	Log-rank		<0.001	<0.001	<0.001
	Wilcoxon		<0.001	<0.001	<0.001
vs. Lrd	Log-rank			0.016	<0.001
	Wilcoxon			0.053	<0.001
vs. Plm	Log-rank				0.186
	Wilcoxon				0.050

Values represent the mean ± SEM (*n* = 20/group)

While the log-rank test reflects mostly the late phase of survival curves, the Wilcoxon signed-rank test reflects the early phase

Abbreviations: *Can* canola oil, *FHCO* fully hydrogenated canola oil, *Lrd* lard, *Plm* palm oil, *SEM* standard error of the mean

### Blood pressure and lipid levels in serum

**Fig. 3:**
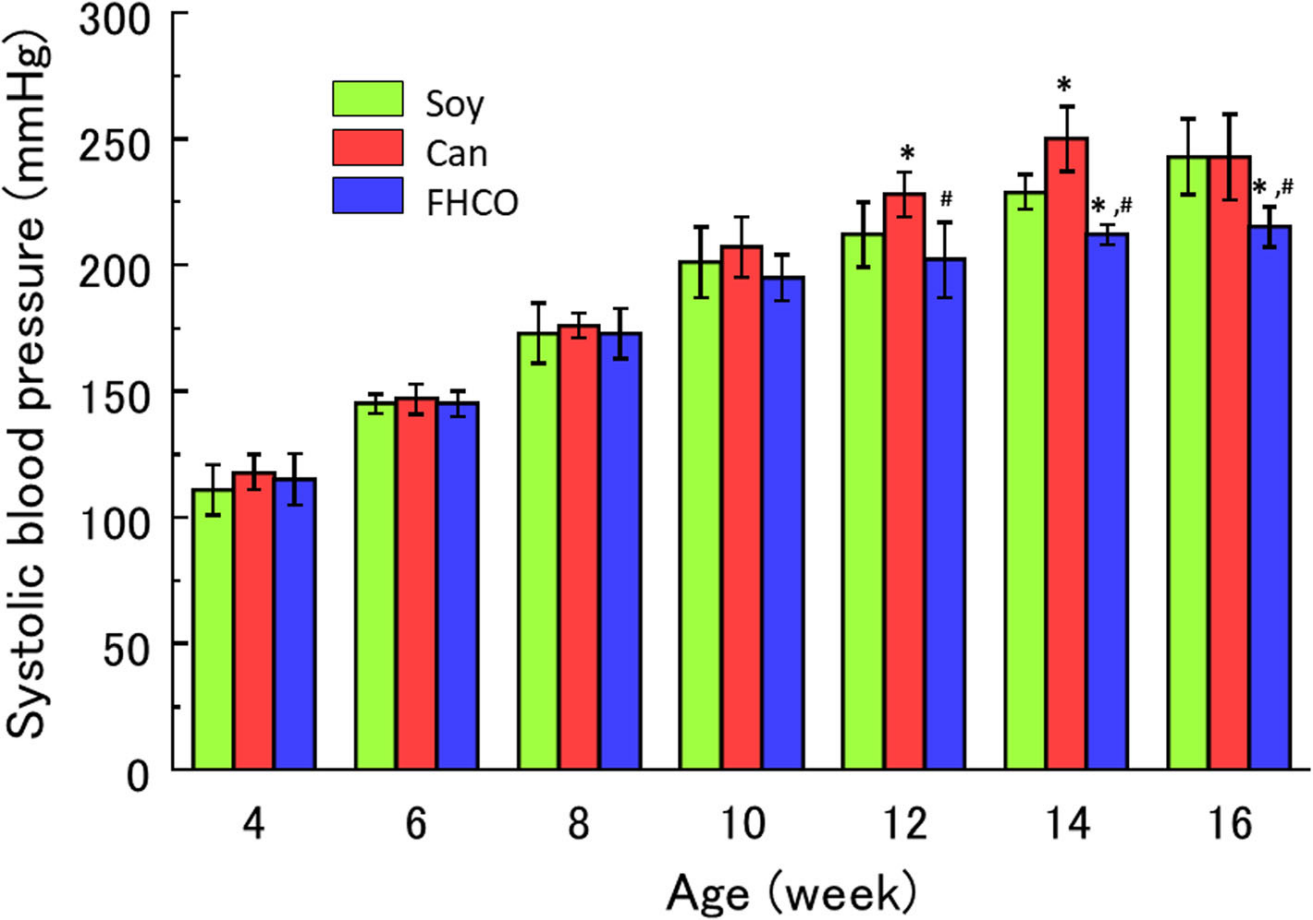
Systolic blood pressure in SHRSP. Values represent mean ± SD (*n* = 12/group from 4 to 11 weeks; *n* = 6/group from 12 to 16 weeks). *P < 0.05 vs. Soy group; ^#^*P* < 0.05 vs. Can group. Abbreviations: Can, canola oil; FHCO, fully hydrogenated canola oil; SD, standard deviation; SHRSP, stroke-prone spontaneously hypertensive rat; Soy, soybean oil

One additional animal experiment was prepared to examine the effects of FHCO on blood pressure, serum lipid levels and pathological conditions. As in the survival tests, FHCO rats in the biochemical analysis test exhibited a decline in the body weight and an increase in food intake relative to other groups although there were no significant differences (Supplementary Fig. S1). None of the rats exhibited any symptoms during the experimental period, except one in the Can group that died at 15 weeks old, probably because of cerebral hemorrhage, which is typical in SHRSP. Blood pressure increased in all rats from 4 to 16 weeks old in all the groups (Fig. 3), with no differences observed until 10 weeks. However, the blood pressure in the FHCO group was 9–11 % lower than that in the Soy group at the ages of 14–16 weeks and 11–15 % lower in the Can group at the ages of 12–16 weeks. Meanwhile, the blood pressure levels were 12 % higher in the Can group than in the Soy group at the ages of 12–14 weeks.

**Fig. 4:**
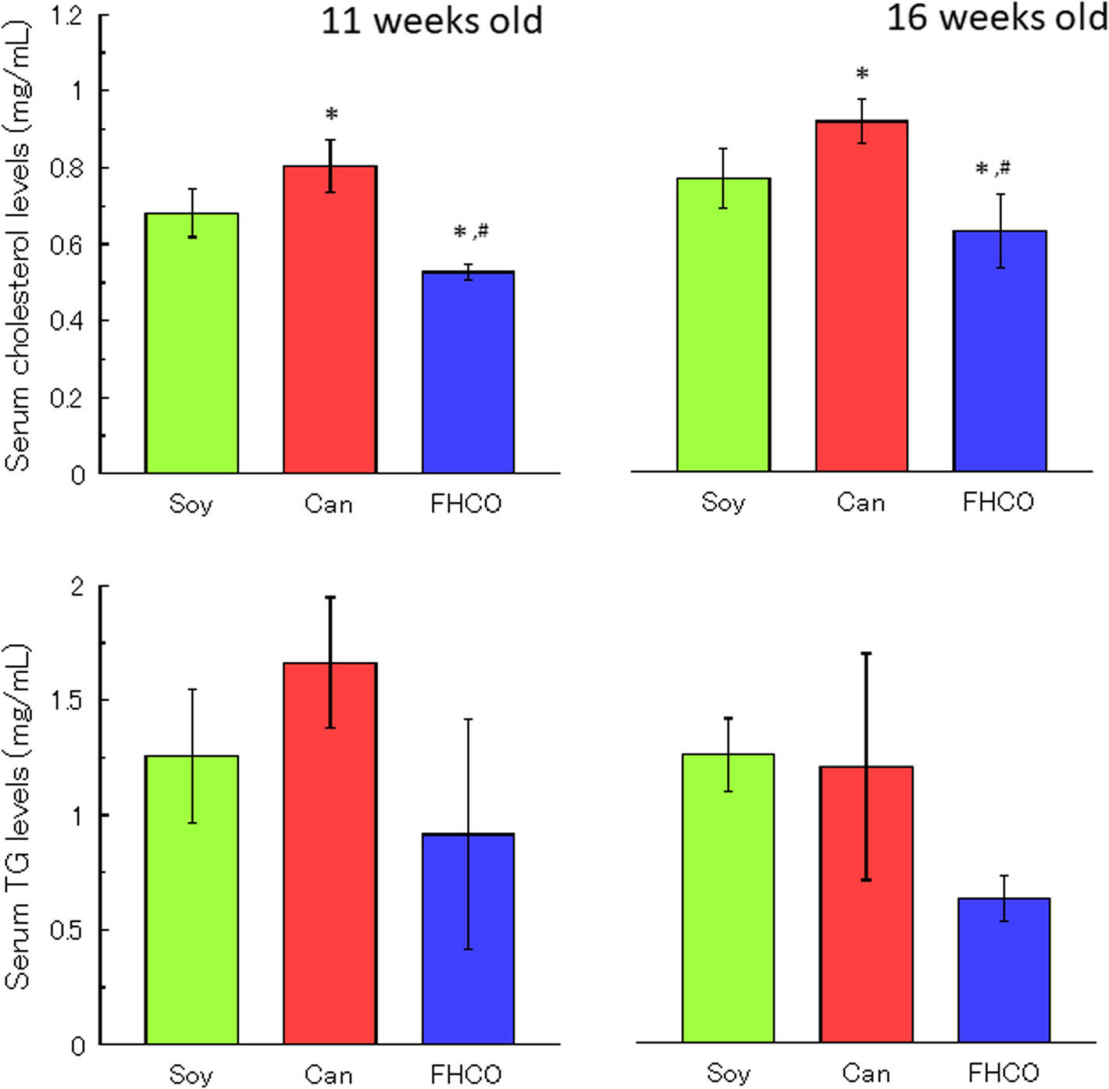
Serum cholesterol and TG levels in SHRSP at (**a**) 11 and (**b**) 16 weeks old. Values represent mean ± SD (*n* = 6/group). *P < 0.05 vs. Soy group; ^#^*P* < 0.05 vs. Can group. Abbreviations: Can, canola oil; FHCO, fully hydrogenated canola oil; SD, standard deviation; SHRSP, stroke-prone spontaneously hypertensive rat; Soy, soybean oil; TG, triacylglycerol

Serum cholesterol and TG levels were examined to determine the effects of dietary oils on the lipid metabolism in SHRSP (Fig. 4). At 11 weeks old, cholesterol levels in the Can group were significantly higher (0.80 ± 0.07 mg/mL) than those in the Soy (0.68 ± 0.06 mg/mL) and FHCO (0.53 ± 0.02 mg/mL) groups. This trend persisted until 16 weeks. In addition, serum TG levels were highest in the Can group (1.70 ± 0.28 mg/mL), followed by the Soy (1.28 ± 0.29 mg/mL) and FHCO (0.93 ± 0.50 mg/mL) groups, although these differences were not statistically significant. At 16 weeks old, the difference in TG levels between the Can and Soy groups disappeared (1.20 ± 0.49 and 1.25 ± 0.16 mg/mL, respectively), whereas the level in the FHCO group remained lower (0.63 ± 0.10 mg/mL).

### Histological analysis

**Fig. 5:**
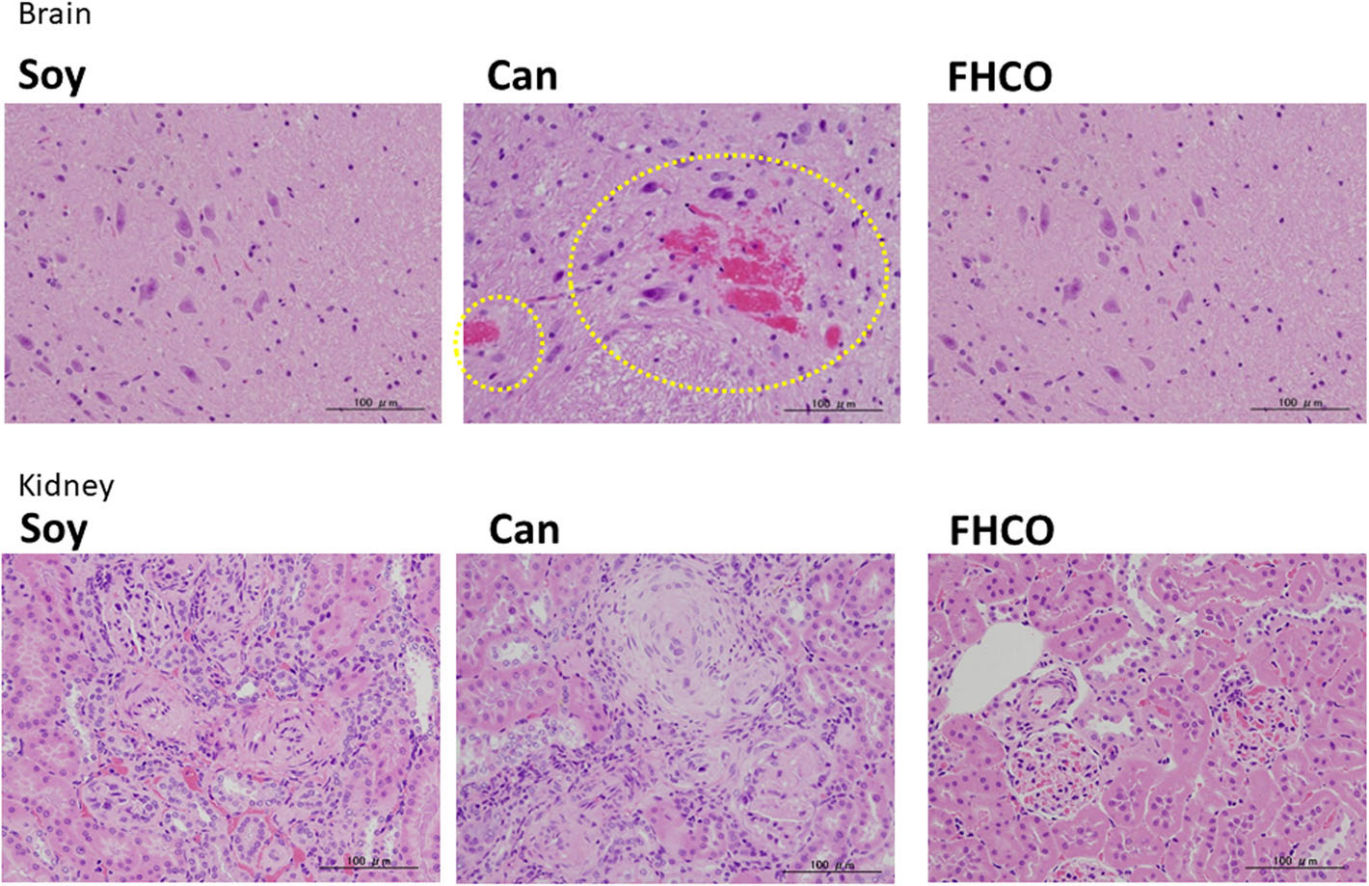
Histological analysis of the brain (top) and kidney (bottom) tissue samples from SHRSP fed Soy (**A**), Can (**B**), and FHCO (**C**) diets at 16 weeks old. The tissue sections were stained with hematoxylin and eosin. Yellow circles indicate the location of hemorrhage. Abbreviations: Can, canola oil; FHCO, fully hydrogenated canola oil; SHRSP, stroke-prone spontaneously hypertensive rat; Soy, soybean oil

Brain and kidney sections stained with hematoxylin and eosin were evaluated to determine whether the FHCO diet prevented lethal stroke in SHRSP (Fig. 5; Table 4). There were no significant differences in weights of either tissue type at 11 and 16 weeks old. At 11 weeks old, none of the rats in the three groups exhibited brain abnormalities. At 16 weeks, 2 of the 5 rats in the Can group exhibited evidence of hemorrhage at the brain cortex and cerebellum medulla, respectively (scored ±); however, no abnormalities were observed in the other two groups. In the kidneys, basophilic tubules and cortex were observed in a few rats from the three dietary groups, but almost none were very severe at 11 weeks old. At the same age, one rat from the FHCO group exhibited marginal mineralization. However, various severe symptoms were observed in the Soy and Can groups, such as basophilic tubules, hyaline casts, glomerular sclerosis, arteriole stenosis, and arterial wall thickening at 16 weeks old. Particularly, all rats from the Can group had these symptoms graded as severe, except mineralization. In the FHCO group, four rats exhibited basophilic tubules and one exhibited hyaline casts, but these symptoms were marginal (only ±) compared with those of the other two dietary groups.

**Table 4:** Summary of the histological analysis of the brain and kidney from SHRSP

	11-week-old			16-week-old		
	Soy (*n* = 6)	Can (*n* = 6)	FHCO (*n* = 6)	Soy (*n* = 6)	Can (*n* = 5)	FHCO (*n* = 6)
**Brain**						
Hemorrhage					(±) × 2	
**Kidney**						
Basophilic tubule, cortex	(±) × 3	(±) × 3, (+) × 3	(±) × 4	(±) × 4, (+) × 1, (⧺) × 1	(⧺) × 5	(±) × 4
Cast, hyaline				(±) × 2	(±) × 1, (+) × 4	(±) × 1
Mineralization			(±) × 1	(±) × 2		
Sclerosis, glomerulus				(±) × 1	(±) × 2, (+) × 3	
Stenosis, arteriole				(+) × 1	(+) × 2, (⧺) × 3	
Thickening, arterial wall				(+) × 1	(+) × 2, (⧺) × 3	

Values represent the grade of incidence × the number of rats

Histology was analyzed using five grades (-, ±, +, ⧺, ⧻) and represents the number scored more than (±)

Abbreviations: *Can* canola oil, *FHCO* fully hydrogenated canola oil, *SHRSP* stroke-prone spontaneously hypertensive rat, *Soy* soybean oil

## Discussion

The dietary intake of Can shortens the lifespan in SHRSP [4, 7, 10, 22, 35, 36], similar to the effect observed in partially hydrogenated oils containing *trans* FA, although the original oil does not exhibit such toxicity [30]. These findings suggest that some toxic substances are produced during the hydrogenation process. In the present study, full hydrogenation appeared to enhance the production of toxic substances and resulted in drastic shortening of the lifespan. However, the findings revealed that full hydrogenation of Can eliminates its lifespan shortening ability (Fig. 2). In addition, the FHCO diet lowered systolic blood pressure and serum cholesterol and TG levels and prevented cerebral hemorrhage and renal dysfunction in SHRSP compared with the other two dietary groups (Figs. 3, 4 and 5). Previously, a diet high in n-3 oil (flaxseed or fish oil) was reported to prolong SHRSP survival [3, 12]. The extremely elevated blood pressure following renal dysfunction causes lethal stroke in SHRSP [37]. Surprisingly, rats fed on an FHCO diet never had lethal cerebral strokes even under 1 % NaCl loading in this study (> 180 days). To the best of our knowledge, this is the first study demonstrating that a lethal stroke in SHRSP could be prevented solely by lipid nutrition, although the underlying mechanisms or substances associated with the beneficial effects are yet to be identified. In addition, the mean survival time of SHRSP fed the Plm diet was marginally extended compared to that of rats fed the Can diet but was markedly shortened compared to those fed the Lrd diet, suggesting that these plant oils (Can and Plm) are toxic for SHRSP and that Plm contains lower levels of toxic substances than Can.

In the present study, body weight gain was suppressed in the Can group starting at 9 weeks old, with a concomitant reduction in food intake (Fig. 1). These findings can be attributed to stroke-related dysfunction in eating, as evidenced by the markedly shortened lifespan accompanied with the earlier onset of stroke (Fig. 2; Table 3). In addition, the FHCO group demonstrated a marked decline in body weight gain starting at 9 weeks old but maintained a higher food intake than the other three groups throughout the study period (Fig. 1). The poor absorption of FHCO could explain these findings in the FHCO group. Essentially, tristearin, a major component of FHCO, is indigestible in rats [38]. Thus, it is conceivable that the food intake in the FHCO group increased to compensate for the resultant nutritional deficiency because of such malabsorption. Furthermore, the light-colored feces observed in this group suggested malabsorption of FHCO, although the fat components in feces were not analyzed in the current study. However, no abnormal sign was noted in the gross observation of animals in the FHCO group compared with any other group examined.

Despite the marked differences in the lifespan, body weight gains and food intakes were similar in the Can and FHCO groups during the 12-week feeding period (Supplementary Fig. S1). In the present study, all rats were loaded with NaCl via drinking water. As NaCl loading facilitates an increase in blood pressure in SHRSP [39], the decreased body weight gain in the Can group could be attributed to exacerbation of hypertension- or stroke-related conditions. According to previous studies, Can shortens the lifespan regardless of NaCl loading [4, 5, 11]. Currently, various edible oils in the market, including linseed oil (n-3 FA), coconut oil (middle-chain FA), and olive oil (polyphenols), are advertised as exerting a slimming effect [40–46]. FHCO may also exhibit a slimming effect due to the limiting lipid absorption.

In the current study, the Can diet elevated blood pressure in SHRSP from 12 to 14 weeks old compared with the control Soy diet (Fig. 3); this is consistent with one prior study [13] but not with another [12]. However, the latter study reported that a Can diet elevated renin levels and promoted renal dysfunction to a greater extent than did other oils [12]. The high blood pressure caused by abnormalities in the renin–angiotensin system resulted in severe renal disorder in spontaneously hypertensive rats [47] or SHRSP [48], indicating that the Can diet may affect the renin–angiotensin system and injure the kidneys, irrespective of changes to blood pressure. Meanwhile, the FHCO diet also decreased blood pressure levels; this reduction was small but may have contributed to the suppression or delay of the onset of cerebral stroke in SHRSP and could be a vital determinant of survival modulated by lipid nutrition. However, these findings contradict epidemiological studies of human nutrition; that is, the dietary intake of saturated fat increases blood pressure in human [49]. Although the underlying mechanism remains unknown, a reduction in blood pressure by FHCO may have relevance in the prevention of lethal cerebral strokes.

Reportedly, SHRSP have low cholesterol levels in the erythrocyte membrane and exhibit an abnormal accumulation of phytosterols, possibly because of dysfunction in the transport through ABCG5/8 by a point mutation in *abcg5/8* [9, 50]. This results in delayed cholesterol supplementation in the event of a lethal stroke in SHRSP and shortens the lifespan [7, 11, 51]. In the present study, the FHCO and Can diets had similar phytosterol compositions (Table 2), indicating that a full hydrogenation reaction did not destroy the conformation of sterols in Can. Although Can-fed SHRSP had shortened lifespan, no FHCO-fed rats died, which supports a previous hypothesis that phytosterol levels in edible oils are not the only factor determining SHRSP survival [11]. In addition, the FHCO diet lowered serum cholesterol and TG levels, thereby reducing the risk of hemodynamic disorders (Fig. 4). Excess SFA intake may limit the effects of polyunsaturated FAs (PUFAs), which serve as ligands for lipid metabolism-related transcription factors, including sterol regulatory element-binding protein (SREBP) and peroxisome proliferator-activated receptor (PPAR). Typically, PUFA deficiency causes upregulation of SREBP and downregulation of PPAR, thus enhancing the expression of lipid synthesis enzymes [52]. This study suggests that the effects of the FHCO diet on serum lipid levels does not depend on the regulation of lipid synthesis, although further confirmation is required by the expression of these transcription factors using quantitative reverse transcription polymerase chain reaction. Of note, a high SFA content may prevent micelle formation, resulting in declined absorption of FA and cholesterol in the intestine; in turn, this poor absorption may affect phytosterol and toxic substance levels in the body. Ergosterol has been shown to regulate SREBP cleavage in fission yeast, and stigmasterol has been shown to activate liver X-receptor in a cell-based reporter assay [53, 54]. Furthermore, various factors attributed to FHCO ingestion may directly or indirectly affect the pathophysiology of SHRSP.

The protective effects of FHCO against stroke in SHRSP may be exerted via various mechanisms. Calorie restriction could be a potential mechanism. Tristearin, which is a major component of FHCO, is poorly absorbed in normal rats [38, 55]. In conjunction with an adequate serving of protein, a 5 % fat or fat-free diet extends the survival of SHRSP compared with a 10 % fat diet [56]. As mentioned earlier, rats fed the FHCO diet exhibited increased food consumption without body weight gain in this study (Fig. 1). These conflicting results could be attributed to the decreased absorption of nutrients, resulting in a delay in weight gain, and a concomitant increase in food intake to compensate for the calorie deficit. Another possible mechanism is conversion of any toxic substances in Can to nontoxic or even beneficial ones in FHCO. Furthermore, it is possible that microbial metabolites affect the physiological condition of SHRSP, because normotensive rats showed elevated blood pressure by gavage with SHRSP’s microbiota [57]. Although the details of molecules formed by complete hydrogenation remain unknown, the existence and roles of some small fatty molecules generated in processed oils should be considered. To date, such ingredients have not been eliminated when discussing the advantages and disadvantages of lipid nutrition.

In the present study, cerebral damage was only observed in two rats, suggesting that cerebral disorder was marginal in the Can group at 16 weeks, although SHRSP died from consumption of under 1 % NaCl (Figs. 2 and 5). This condition could represent the physiological state that easily induced stroke in Can-fed SHRSP, resulting in cerebral disorder due to other factors, such as NaCl loading. Conversely, kidneys from SHRSP exhibited apparent differences between the three dietary groups. The findings revealed that renal dysfunction in the Can group resulted in elevated blood pressure (Fig. 3). These blood pressure increases from the renin–angiotensin system may be compounded by effects on the kidneys, with the resulting elevation in blood pressure playing a crucial role in the determination of the pathological condition of SHRSP.

### Comparisons with other studies and what does the current work add to the existing knowledge

In the current study, nutrition was evaluated based on the “lifespan” of specific pathological animal models. There are a few reports of prolonged survival time of SHRSP after use of dietary oils (perilla oil or fish oil), but almost all rats died in those studies [3, 10]. Here, the FHCO diet repaired the pathological condition of SHRSP, and no rats died within the experimental period. Presumably, this is the first study to demonstrate that a lethal stroke in SHRSP can prevented solely by lipid nutrition. The Can diet elevated blood pressure and serum lipid levels, shortening the survival of SHRSP. It is also the first study to report the effect of Plm on the survival of SHRSP.

### Study strength and limitation

In SHRSP, the FHCO diet prevented lethal cerebral stroke, limited increase in blood pressure, lowered serum lipid levels, cured renal dysfunctions, and so on. It is impressive that the specific pathological condition of SHRSP was changed with only lipid nutrition. Further, conflicting findings regarding food intake and body weight were observed, suggesting the possibility of using FHCO to regulate body weight. From another perspective, original Can diet shortened the lifespan, but this toxic effect was absolutely inverted by full hydrogenation. This result might provide key evidence for determining the unknown factor(s) that shorten the lifespan in SHRSP. However, these results were gained from specific animal models. Therefore, it is difficult to know which effects of FHCO are associated with either human or other animal species. The underlying mechanisms or substances associated with the beneficial effects are yet to be identified. Further studies are now required to elucidate the possible applications of FHCO.

## Conclusions

The present study demonstrated that the FHCO diet suppresses lethal cerebral stroke in SHRSP, in addition to limiting blood pressure elevation, decreasing serum lipid levels, and preventing the progression of pathological symptoms. In addition, FHCO prevents body weight gain despite increased food intake. However, the current study confirmed the toxicity of Can, which elevates blood pressure and lipid levels, thereby accelerating the onset of stroke. These results demonstrate that lipid nutrition improves or worsens the physiological state of SHRSP, and may provide a basis for the development of new approaches to lipid nutrition, including for therapeutic applications.

## Supplementary Information

**Additional file 1: Supplementary Table S1.** Statistical analysis of the first abnormal incidence. Values represent the mean ± SEM (*n* = 20/group). Abnormal incidence was the following; visible bleeding, diarrhea, and paralysis. While the log-rank test reflects mostly the late phase of survival curves, the Wilcoxon signed-rank test reflects the early phase. Abbreviations: Can, canola oil; FHCO, fully hydrogenated canola oil; Lrd, lard; Plm, palm oil; SEM, standard error of the mean.

**Additional file 2: Supplementary Figure S1.** (a) Body weight and (b) food intake in SHRSP. Values represent mean ± SD (*n* = 6–12/group for body weight and *n* = 4/group for food intake). *P < 0.05 vs. Soy group; ^#^*P*< 0.05 vs. Can group. Abbreviations: Can, canola oil; FHCO, fully hydrogenated canola oil; SHRSP, stroke-prone spontaneously hypertensive rat; Soy, soybean oil.

## Abbreviations

abcg5/8: ATP-binding cassette transporter G5/8
ANOVA: Analysis of variance
Can: Canola oil
FA: Fatty acid
FHCO: Fully hydrogenated canola oil
Lrd: Lard
Plm: Palm oil
PPAR: Peroxisome proliferator-activated receptor
PUFA: Polyunsaturated fatty acid
SFA: Saturated fatty acid
SHRSP: Stroke-prone spontaneously hypertensive rat
Soy: Soybean oil
SREBP: Sterol regulatory element-binding protein
TG: Triacylglycerol
