# Supplementary material for: Fully hydrogenated canola oil extends lifespan in stroke-prone spontaneously hypertensive rats
Source: Lipids Health Dis. 2021 Sep 12;20:102. doi: 10.1186/s12944-021-01540-7 (PMC8436556; doi:10.1186/s12944-021-01540-7)
Supplement: Supplementary file 1 — Additional file 1: Supplementary Table S1. Statistical analysis of the first abnormal incidence. Values represent the mean ± SEM (n = 20/group). Abnormal incidence was the following; visible bleeding, diarrhea, and paralysis. While the log-rank test reflects mostly the late phase of survival curves, the Wilcoxon signed-rank test reflects the early phase. Abbreviations: Can, canola oil; FHCO, fully hydrogenated canola oil; Lrd, lard; Plm, palm oil; SEM, standard error of the mean. [file 12944_2021_1540_MOESM1_ESM.docx]

**Supplementary Table S1**  Statistical analysis of the first abnormal incidence

|  | **Statistical test** | **FHCO** | **Lrd** | **Plm** | **Can** |
| --- | --- | --- | --- | --- | --- |
| Age of  (mean ± SEM) |  | > 180 ± 0 | 107 ± 7 | 97 ± 3 | 88 ± 3 |
| vs. FHCO | Log-rank |  | <0.001 | <0.001 | <0.001 |
|  | Wilcoxon |  | <0.001 | <0.001 | <0.001 |
| vs. Lrd | Log-rank |  |  | 0.069 | 0.005 |
|  | Wilcoxon |  |  | 0.321 | 0.013 |
| vs. Plm | Log-rank |  |  |  | 0.055 |
|  | Wilcoxon |  |  |  | 0.003 |

Values represent the mean ± SEM (*n* = 20/group).

Abnormal incidence was the following; visible bleeding, diarrhea, and paralysis.

While the log-rank test reflects mostly the late phase of survival curves, the Wilcoxon signed-rank test reflects the early phase.

Abbreviations: Can, canola oil; FHCO, fully hydrogenated canola oil; Lrd, lard; Plm, palm oil; SEM, standard error of the mean.
