# Supplementary figures and images for: Fully hydrogenated canola oil extends lifespan in stroke-prone spontaneously hypertensive rats
Source: Lipids Health Dis. 2021 Sep 12;20:102. doi: 10.1186/s12944-021-01540-7 (PMC8436556; doi:10.1186/s12944-021-01540-7)

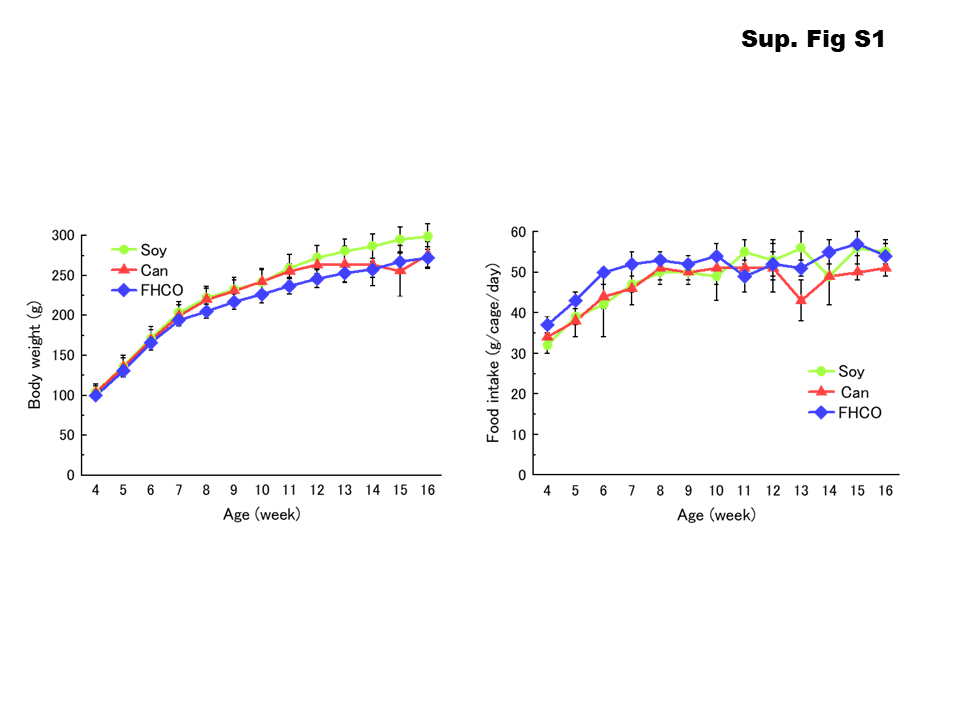

Supplement: Supplementary file 2 — Additional file 2: Supplementary Figure S1. (a) Body weight and (b) food intake in SHRSP. Values represent mean ± SD (n = 6–12/group for body weight and n = 4/group for food intake). *P < 0.05 vs. Soy group; #P< 0.05 vs. Can group. Abbreviations: Can, canola oil; FHCO, fully hydrogenated canola oil; SHRSP, stroke-prone spontaneously hypertensive rat; Soy, soybean oil. [file 12944_2021_1540_MOESM2_ESM.tif]
